# Study of Hypoglycemic Activity of Novel 9-N-alkyltetrahydroberberine Derivatives

**DOI:** 10.3390/ijms232214186

**Published:** 2022-11-16

**Authors:** Mikhail V. Khvostov, Elizaveta D. Gladkova, Sergey A. Borisov, Marina S. Fedotova, Nataliya A. Zhukova, Mariya K. Marenina, Yuliya V. Meshkova, Olga A. Luzina, Tatiana G. Tolstikova, Nariman F. Salakhutdinov

**Affiliations:** 1 N. N. Vorozhtsov Novosibirsk Institute of Organic Chemistry, Siberian Branch of the Russian Academy of Sciences, 9, Akademika Lavrentieva Ave., 630090 Novosibirsk, Russia; 2 Department of Natural Sciences, Novosibirsk State University, Pirogova Str. 1, 630090 Novosibirsk, Russia; 3 V. Zelman Institute for the Medicine and Psychology, Novosibirsk State University, Pirogova Str. 1, 630090 Novosibirsk, Russia

**Keywords:** berberine derivatives, metabolic syndrome, hypoglycemic activity, OGTT, agouti yellow mice

## Abstract

Novel 9-N-alkyltetrahydroberberine derivatives were synthesized, among which, based on the results of OGTT, one compound containing the longest aliphatic substituent was selected for study in mice C57BL/6^Ay^, which demonstrate obesity, impaired glucose tolerance, and concomitant liver non-alcoholic fatty disease. Administration of this substance at a dose of 15 mg/kg for four weeks improved the insulin sensitivity of mice, which resulted in a decrease in fasting glucose levels and improved the tolerance of mice to OGTT glucose loading. A decrease in the level of lactate in the blood and a decrease in the amount of glucokinase in the liver were also found. The introduction of compound 3c did not have a toxic effect on animals based on biochemical data, histological analysis, and measurements of general parameters such as body weight and feed intake. Thus, the 9-N-heptyltetrahydroberberine derivative showed prominent hypoglycemic effects, which makes it promising to obtain and study other derivatives with longer substituents.

## 1. Introduction

Diabetes mellitus is a complex and heterogeneous disease that can affect people at different life stages. This disease is characterized by an impairment of carbohydrate, lipid, and protein metabolism. Together, they lead to an impairment of insulin secretion by pancreatic beta cells and/or the emergence of insulin resistance in insulin-dependent tissues: skeletal muscle, liver, and fat. Among the three known types of diabetes mellitus, Type 2 diabetes mellitus (T2DM) is the most common and is often referred to as an epidemic affecting developed countries of the world and is a noticeable disorder in the structure of metabolic syndrome [1]. Correction of this pathology begins with a change in lifestyle and diet. When this becomes ineffective, drug therapy comes into play. For many years, the first line of therapy has been metformin (MF), whose known mechanisms of action include a decrease in gluconeogenesis in the liver, stimulation of glucose intake in muscles, and an increase in fatty oxidation in adipose tissues [2]. However, it is poorly tolerated by many patients, in particular due to gastrointestinal adverse effects [3], which requires its replacement by other hypoglycemic agents. In addition, comorbidities also affect the choice of a more effective and safer drug. Current drug therapy, in addition to metformin, may include drugs from the following pharmacological classes: Sulfonylureas, Glinides, Thiazolidinediones, Dipeptidyl peptidase-4 (DPP-4) inhibitors, Glucagon-like peptide-1 (GLP-1) receptor agonists, alpha glucosidase inhibitors, sodium glucose co-transporter-2 (SGLT2) inhibitors [4]. However, all of the above drugs exhibit various undesirable side effects [5], which limits their application. Thus, the growing problem of T2DM is the lack of an ideal drug solution, which stimulates research and development of new drugs, including those with new mechanisms of action.

**Figure 1:**
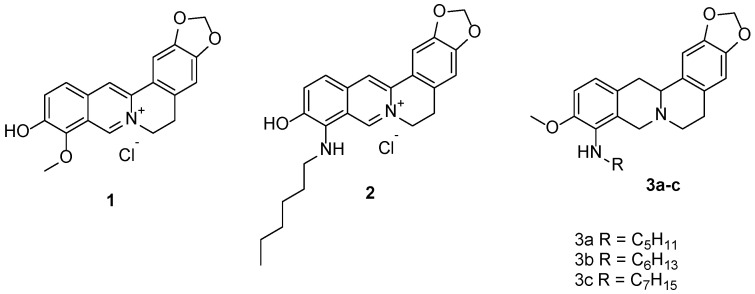
Structures of berberine **1**, 9-N-hexylberberine **2**, and 9-N-alkyltetrahydroberberines **3a**–**c**.

Berberine 1 (Figure 1) is an isoquinoline plant alkaloid, long known to Chinese folk medicine [6,7,8]. Among the various pharmacological properties, one of the most significant is its hypoglycemic action [9,10], the exact mechanism of which is still not fully understood. Nevertheless, the known molecular mechanisms of its pharmacological action allow us to consider it a multitarget agent. Such mechanisms include α-glucosidase inhibition [11], regulation of the peroxisome proliferator-activated receptors [12,13], and the expression of positive transcription elongation factor b in diabetic adipocytes [13], gluconeogenesis inhibition in the liver [14], modulation of the farnesoid X receptor (FXR) signaling pathway in the intestine [15,16], inhibition of the lipopolysaccharide (LPS)-induced toll-like receptor (TLR)4/tumor necrosis factor (TNF)-α activation [17], activation of an AMP-activated protein kinase (AMPK) pathway [18,19]. In addition, berberine is able to activate peroxisome proliferator-activated receptor (PPAR)-γ and inhibit protein tyrosine phosphatase 1B (PTP1B) activity [20]. However, from a drug point of view, berberine is not a very good molecule due to the extremely low oral bioavailability (~1%) [21], which requires the use of high doses (380 mg/kg) [22]. In addition, berberine has a pronounced antibacterial effect, which, with prolonged use, will have a negative impact on the microbiome of the gastrointestinal tract [23], and may affect endocrine factors [24]. The sub-acute concentrations of berberine lead to altered liver function, gastric troubles, hepato and hematotoxicity, hemorrhagic inflammatory consequences, and damage to immune cells [25].

As described in [26], modification of the berberine molecule by the 9-position is the most promising direction for obtaining derivatives with a pronounced hypoglycemic effect. On this basis, we previously obtained 9-hexylaminoberberine 2 [27], which showed a pronounced hypoglycemic effect at a dose of 15 mg/kg both in an oral glucose tolerant test (OGTT) by single administration and in a long-term experiment on C57BL/6^Ay^ mice (AY mice), which demonstrate obesity, impaired glucose tolerance, and concomitant non-alcoholic fatty liver disease. One of the findings of this study was a very prominent blood glucose decrease by compound **2**, which caused severe hypoglycemia [27]. Continuing the study of this type of substance and based on evidence that the hydrogenated form is more bioavailable [19], we obtained the hydrogenated form of this compound and its closest analogues (C5, C7) in order to study the effect of this modification on their hypoglycemic effect, which all together became the basis for this article.

## 2. Results

### 2.1. Synthesis

**Scheme 1:**
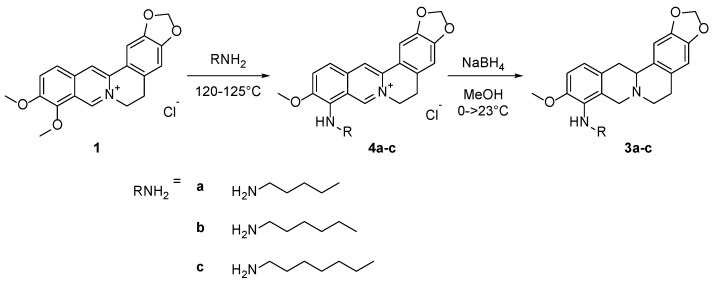
Synthetic route for preparing compounds **3a**–**c** from berberine **1**.

The tetrahydroderivatives **3a**–**c** were synthesized in two steps from berberine **1** (Scheme 1). First, we heated berberine with the corresponding amine, which resulted in the intermediate products **2a**–**c**. After reduction of these compounds, **2a**–**c**, by NaBH_4_ key products, **3a**–**c** were formed and purified by colon chromatography. Total yield of the products after two steps varied from 25 to 45% depending on the substrate. 

### 2.2. OGTT Screening

Compounds **3a**–**c** were evaluated for their hypoglycemic activity at several doses. The initial dose was 15 mg/kg. Compound **3a** was found to have no hypoglycemic effect. Compound **3b** had a very pronounced effect at a dose of 30 mg/kg, which resulted in fatal hypoglycemia in 2 mice, but showed a moderate effect at a dose of 15 mg/kg. Compound **3c** showed the broadest range of effective doses and even at a dose of 45 mg/kg did not cause as much fatal hypoglycemia as compound **3b** at a dose of 30 mg/kg (Table 1). Based on these results, compound **3c** at a dose of 15 mg/kg was chosen for a subsequent experiment on AY mice.

### 2.3. Body Weight and Food Consuption

**Figure 2:**
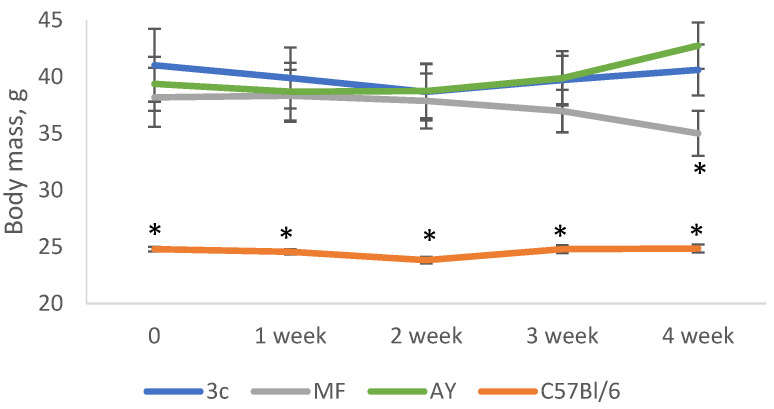
Body mass change during the experiment. * *p* < 0.05 as compared to AY mice.

During the experiment, animals in all groups showed similar change in body weight, with the exception of mice treated with MF. In the MF group, animal weight by the end of the experiment was the lowest among AY mice (Figure 2).

The dynamics of feed consumption by mice in all experimental groups differed from each other (Table 2). A decrease in this index was noted in animals receiving MF. The mice that received compound **3c** generally consumed the same amount of feed throughout the experiment, which, together with the dynamics of body weight of these animals, indicates the absence of toxicity of this substance.

### 2.4. OGTT after 14 Days of Experiment

**Figure 3:**
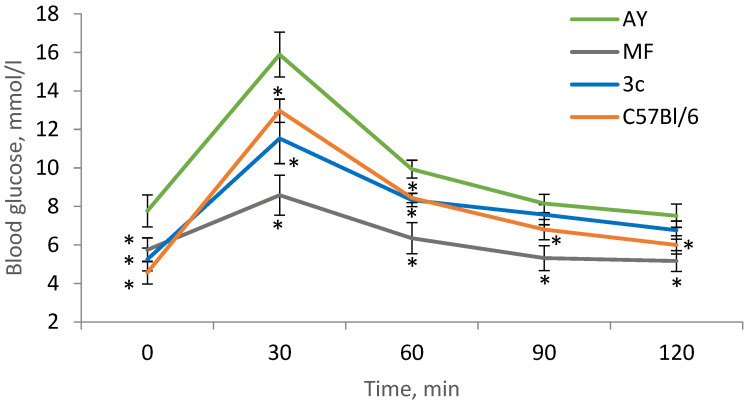
The results of the OGTT performed after 2 weeks of AY mice treatment by compound **3c** at a dose of 15 mg/kg. MF was introduced at a dose of 250 mg/kg. * *p* < 0.05 as compared to AY mice.

Hypoglycemic effect of compound **3c** on glucose load after two weeks was shorter and less pronounced than that of the comparison drug metformin (Figure 3). Initial glucose levels were lower in both groups compared to untreated AY mice.

### 2.5. OGTT after 28 Days of Experiment

**Figure 4:**
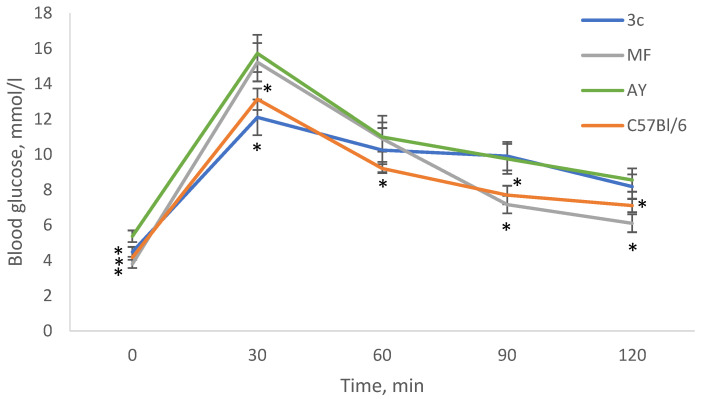
The results of the OGTT performed after 4 weeks of AY mice treatment by compound **3c** at a dose of 15 mg/kg. MF was introduced at a dose of 250 mg/kg. * *p* < 0.05 as compared to AY mice.

Twenty-eight days after the beginning of the experiment, OGTT was carried out, in which the studied substances were not administered immediately before the test, and the last introduction was made ~24 h before the glucose load. In this case, the cumulative effect of the studied substances on the carbohydrate metabolism of the animals was evaluated. According to the test results, compound **3c** and MF were found to have different hypoglycemic effects. In mice treated with compound **3c**, the glucose level after 30 min was much lower than in mice from the negative control and MF groups (Figure 4). In contrast, MF administration contributed to lower glucose levels at 90 and 120 min after the start of the test. Initial glucose levels were lower in both groups compared to untreated AY mice.

**Figure 5:**
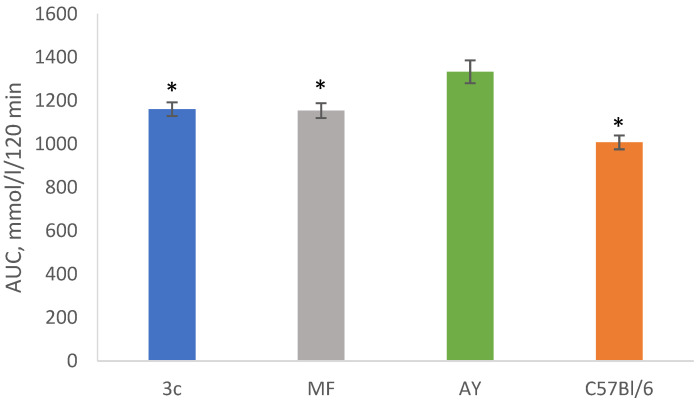
Area under the glycemic curve calculated according to the OGTT data after 4 weeks of AY mice treatment by compound **3c** at a dose of 15 mg/kg and MF at a dose of 250 mg/kg. * *p* < 0.05 as compared to AY mice.

When evaluating the area under the glycemic curve, the effects of compound **3c** and MF were the same (Figure 5).

### 2.6. Insulin Tolerance Test (ITT) after 29 Days of Experiment

**Figure 6:**
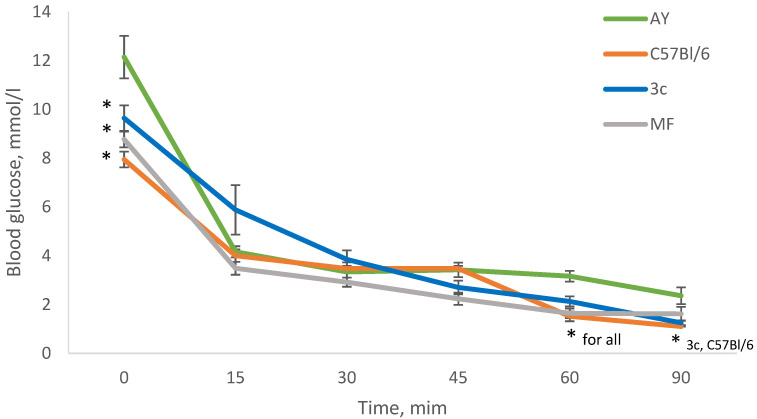
Blood glucose levels in ITT performed 29 days after the beginning of the experiment. * *p* < 0.05 as compared to AY mice.

After 29 days from the beginning of the experiment, the insulin sensitivity of the mice was evaluated, and it was found that compound **3c** significantly increases it. This is shown in Figure 6, time points of 60 and 90 min. It is worth noting here that the glucometer test strips used have a lower threshold of 1.1 mmol/L, which did not allow the true values to be assessed. Mice in the compound **3c** group most likely had glucose levels below this threshold, since they showed signs of severe hypoglycemia: decreased mobility, decreased body temperature, and sometimes convulsions. All mice in the compound **3c** group were immediately injected with a 10% glucose solution (0.5 mL/mouse) intraperitoneally when these symptoms appeared. This prevented animal death. A similar pattern was observed in C57Bl/6 mice, a healthy control. In AY mice in the control group, blood glucose levels did not fall to such low values. No signs of sever hypoglycemia were observed and, accordingly, no glucose was injected intraperitoneally at the end of the test. In animals that received MF, the blood glucose concentration decrease after insulin injection was at an intermediate level if compared to the compound **3c** and AY control groups. Their average glucose level at the end of the test was slightly higher than that of compound **3c** and C57Bl/6 mice, but in half of the animals it was at the 1.1 mmol/L level, which was the indication for intraperitoneal glucose administration.

### 2.7. Weight of Organs and Tissues

When measuring the weight of the organs taken at the end of the experiment, it was found that the administration of compound **3c** and MF reduced the mass of brown interscapular fat. Other parameters were similar to those of mice in the AY control group (Table 3).

### 2.8. Biochemical Blood Test

Biochemical examination of the blood of the animals showed that administration of compound **3c** and MF resulted in a decrease in lactate levels, whereas the other measured parameters were not different from AY control mice (Table 4). The absence of changes in liver transaminases and total protein levels indicate the absence of hepatotoxicity of compound **3c** when administered for 30 days at a dose of 15 mg/kg.

### 2.9. ELISA Data

**Figure 7:**
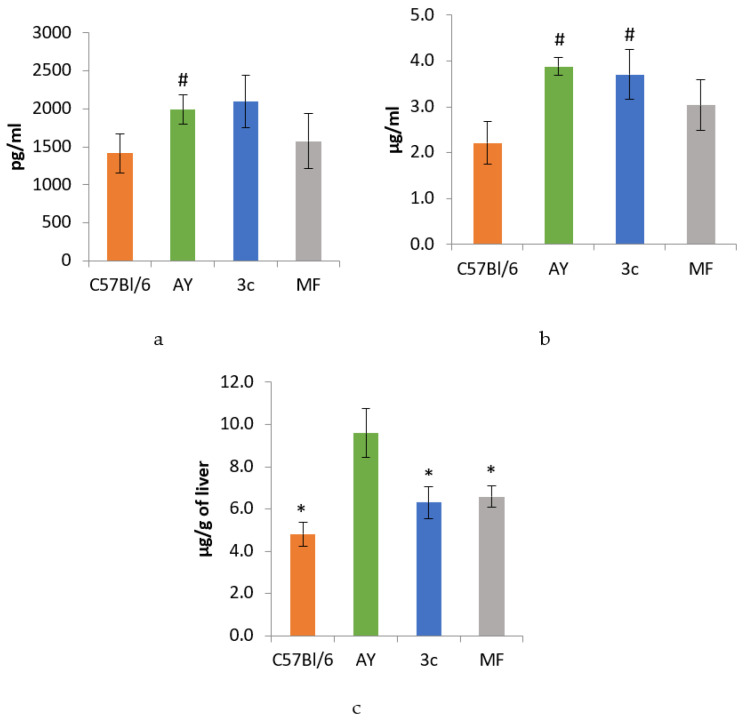
ELISA results. (**a**) Insulin levels in plasma of mice at the end of the experiment. (**b**) Level of adiponectin in plasma of mice at the end of the experiment. (**c**) The amount of glucokinase in the liver of mice at the end of the experiment. * *p* < 0.05 as compared to AY mice; # *p* < 0.05 as compared to C57Bl/6 mice.

The concentrations of insulin and adiponectin in the blood of the animals were determined by ELISA, and the amount of glucokinase was examined in liver homogenates. It was found that the concentration of insulin in the blood of AY mice was higher than that of C57Bl/6 mice (Figure 7a), which corresponds to the literature data [28]. Administration of the studied substances did not change this parameter (Figure 7a). The amount of adiponectin in the blood was also higher in all AY mice (Figure 7b). Glucokinase in the liver was sufficiently reduced in mice treated with compound **3c** and MF (Figure 7c).

### 2.10. Histology

**Figure 8:**
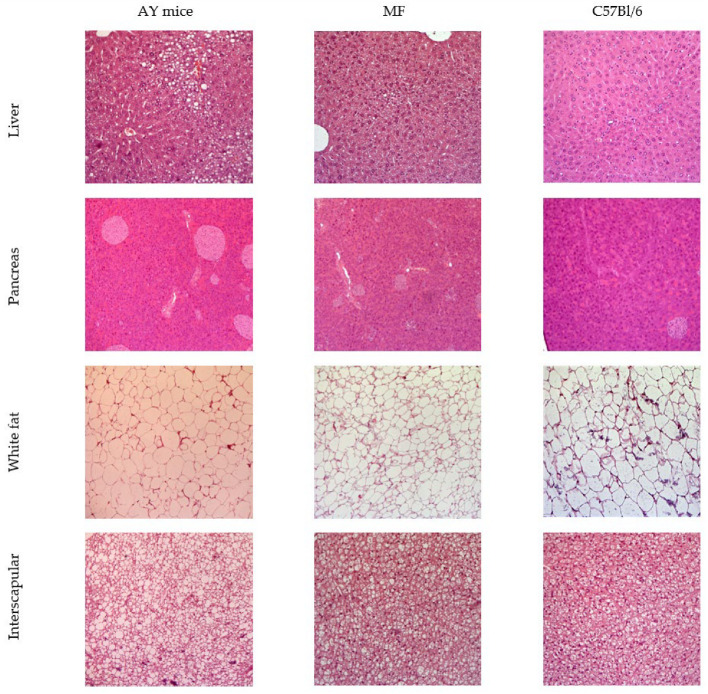
Micrographs of sections of the liver, pancreas, white and brown interscapular fat at the end of the experiment. In case of C57Bl/6 mice, gonadal white fat was investigated. Stained with hematoxylin and eosin. Magnification—liver ×200, other ×100.

AY mice showed degenerative and necrotic changes in the liver. All animals showed dystrophic changes in the form of polymorphic lipid infiltration, focal necrosis of hepatocytes infiltrated by macrophages and mononuclear leukocytes, and hepatic bar dyscomplexation was observed in periportal areas (Figure 8). Numerous enlarged Kupffer cells were detected in the lumen of sinusoids. Glycogen was not detected in the liver during Periodic acid–Schiff staining (PAS staining). Thus, the development of fatty hepatosis was noted in these animals. In the exocrine part of the pancreas, no pronounced alterations (dystrophy, necrosis) were detected. In the endocrine part, there was pronounced hyperplasia of islet apparatus (Figure 8). In the brown adipose tissue, there was a marked increase in adipocyte fat content. Large fat droplets merged with each other to form fat cysts (Figure 8). White adipose tissue also showed a dramatic increase in the size of adipocytes and their fusion into fat cysts (Figure 8). Mice treated with metformin showed improvements in metabolic abnormalities. In the liver, there was a decrease in the severity of dystrophic changes. Small vesicular lipid infiltration was predominantly located in the periportal areas. Inflammatory-necrotic, hemodynamic changes were weakly expressed (Figure 8). Glycogen was detected in the form of dust-like granularity in hepatocytes of the central zones when stained by the PAS method. In sinusoids, there were a large number of PAS-positive macrophages. Focal fatty dystrophy of acinocytes was detected in the exocrine part of the pancreas. In the endocrine part, a decrease in the diameter of the islet apparatus was observed (Figure 8). Under the influence of metformin, fat content in the brown and white adipose tissue also decreased: small drops of fat predominated in adipocytes (Figure 8). Compound **3c** administration had no significant effect on the morphology of the liver and adipose tissue of the animals.

## 3. Discussion

In this study, 9-N-alkyltetrahydroberberines with different length of the aliphatic fragment were synthesized. In this series of compounds, the influence of the length of this fragment on the hypoglycemic property of the molecule can be clearly traced. Thus, in the presence of the pentyl group (compound **3a**), there was no hypoglycemic effect in OGTT. Adding one methylene group (compound **3b**) resulted in the appearance of a marked effect at the dose of 30 mg/kg leading to a fatal drop in the blood glucose level in several mice in the group. It is known that a similar compound with parent berberine skeleton exhibits greater efficiency, markedly reducing the blood glucose level already at a dose of 15 mg/kg [27], which suggests the involvement of the charged moiety of the molecule in the interaction with the pharmacological target. The heptyl substituent (compound **3c**) gives the molecule greater hypoglycemic efficacy with increased safety, namely increasing the dose at which excessive blood glucose reduction occurs in mice (Table 1). Based on these results, compound **3c** was chosen for study of its hypoglycemic action in AY mice. These mice develop yellow pigmentation, late-onset obesity, and hyperinsulinemia, as a result of antagonism of melanocortin receptors by the agouti protein due to mutation of the agouti gene (Ay/a) [28], which makes them a convenient model for studying hypoglycemic effects along with the ability to assess effects on lipid metabolism. In our experiment, AY mice exhibited all of their major features of obesity, hyperinsulinemia, and hyperglycemia (Figure 2, Figure 3, Figure 4 and Figure 7a; Table 3 and Table 4). The safety of compound **3c** at a dose of 15 mg/kg was confirmed in the experiment, as evidenced by the dynamics of animal body weight, which corresponded to that in the negative control group (AY mice, Figure 2). Feed intake of the animals during the experiment decreased only in the metformin group, which correlates with the decrease in the weight of the mice by the end of the experiment (Figure 2, Table 2). In addition, the biochemical parameters of liver condition—ALT, AST, and TP—did not differ from those of the control group mice (Table 4).

Hypoglycemic efficacy of compound **3c** was already clearly observed after two weeks of the experiment, fasting glucose levels were significantly lower than in AY mice from the negative control group, and mice coped better with the oral glucose load in the OGTT (Figure 3). Even more remarkable data were obtained in the OGTT on day 28 of the experiment, when no substance was administered to the animals before the test, but the cumulative effect of the administration was evaluated. In this case, fasting glucose was also lower in the compound **3c** and MF groups compared to AY mice, but the dynamics of the glycemic curve differed in the mice of these three groups (Figure 4). Compound **3c** administration promoted a decrease in peak glucose concentration 30 min after glucose load, which may be responsible for increased insulin release and/or increased tissue sensitivity to insulin. Metformin had a greater effect on late glucose reduction, which may be mediated by skeletal muscle uptake [2]. The area under the glycemic curve was equally smaller in both groups compared with the negative control AY mice (Figure 5). The fact that compound **3c** administration increases the insulin sensitivity of mice is clearly demonstrated by the results of ITT, where there was the most pronounced decrease in glucose among AY mice (Figure 6), as well as a decrease in lactate levels (Table 4). Lactate in large quantities is synthesized by fat cells in obesity, and its elevated blood levels are associated with insulin resistance and, therefore, its reduction can be seen as further evidence of improved tissue sensitivity to insulin [29]. Reduced fasting glucose levels in mice treated with compound **3c** and MF correlate with reduced levels of glucokinase in the liver, indicating a slower rate of glucose deposition [30]. We also measured the level of adiponectin in the blood of the animals and found that it was higher in all AY mice than in C57Bl/6 mice, regardless of the group (Figure 7b). Its concentration correlated directly with body weight and adipose tissue (Figure 2, Table 3). Adiponectin is synthesized by adipocytes and its concentration is usually decreased in obesity, which is considered as one of the causes of insulin resistance. In our case, on the contrary, its concentration was higher, which is explained by the peculiarity of the Agouti yellow mouse line and their insensitivity to adiponectin due to disruption of the melanocortin pathway [28].

Lipid metabolism in AY mice in the experiment changed in the form of an increase in TG level, an increase in the mass of gonadal fat, white and brown interscapular fat (Table 3 and Table 4). The administration of compound **3c** and MF did not affect blood TG levels, but it did affect adipose tissue mass. There was a trend toward decreased interscapular fat mass. The mass of interscapular brown fat in these groups was significantly lower than in untreated AY mice (Table 3). This is associated with a decrease in adipocyte size due to a decrease in lipid droplet size and indicates its activation by stimulating PPAR-α [31]. A similar effect was demonstrated earlier for another derivative of 9-N-hexylberberberine, having a 6-member aliphatic substituent and a charged nitrogen atom in [27].

Histomorphological studies showed the development of fatty hepatosis in AY mice and hyperplasia of the pancreatic islet apparatus. Administration of MF had a positive effect on these organs, reducing the severity of fatty dystrophy and hyperplasia of the pancreatic islet apparatus. In addition, MF contributed to a decrease in the size of fat droplets in the white and brown interscapular fat, which corresponds to a decrease in their mass (Table 3). In mice treated with compound **3c**, the decrease in adipose tissue mass was somewhat less pronounced and, accordingly, no differences from the negative control could be detected on morphological examination. Moreover, compound **3c** administration did not affect the development of fatty liver hepatosis but did not exacerbate it, thus showing no toxic effects on the liver. Despite improved insulin sensitivity, no improvement in pancreatic islet apparatus was noted during compound **3c** administration, which, however, is consistent with blood insulin concentration. Although its concentration did not differ significantly between the groups, in mice treated with MF it tended to decrease (Figure 7a), which was confirmed by pancreatic islet apparatus morphology examination.

If we speculate about the mechanisms of action of compound **3c**, we can state that, mainly, the targets are receptors involved in carbohydrate metabolism, in contrast to MF for which it is known that it can increase fatty oxidation in adipose tissues and is able to reduce body weight and lipid synthesis [2]. Perhaps due to the decrease in brown fat mass, there is some activation of PPAR-α, but judging by the histology data, it is not pronounced enough, which may be due to the low dose of compound **3c** among other things. Many molecular targets than can provide hypoglycemic effect are known. For berberine itself, various mechanisms of action are described in the literature, including the regulation of peroxisome proliferator-activated receptors and the expression of positive transcription elongation factor b [12,13], inhibition of gluconeogenesis in the liver, modulation of the FXR signaling pathway in the intestine [15,16], inhibition of the LPS-induced TLR4/TNF-α activation, which enhances insulin receptor expression in the liver [17], activation of AMPK pathways and inhibition of the α-glucosidase enzyme [18,19]. Moreover, berberine inhibits PTP1B activity [20]. It can be assumed that it is the potent inhibition of PTP1B by tested compounds that is responsible for the death of mice from hypoglycemia in OGTT, as it leads to increased activity of insulin receptors and as a consequence a greater capture of glucose by cells [32].

Summarizing the experimental data obtained, we can state that compound **3c** shows promising properties as a hypoglycemic agent, but it is interesting to continue studying 9-N-alkyltetrahydroberberines with longer aliphatic substituents. If the trend observed in this work continues, the new derivatives may have a wider range of hypoglycemic doses and will be safer in terms of the development of severe hypoglycemia.

## 4. Materials and Methods

### 4.1. Chemistry

^1^H and ^13^C NMR spectra were acquired on Bruker spectrometer AV-400 (Bruker Corporation, Billerica, MA, USA) at 400.13 MHz (^1^H) and 100.61 MHz (^13^C). Spectra were recorded in deuterated chloroform (CDCl_3_); residual CDCl_3_ was used as a standard [δ(CDCl_3_) 7.24, δ(CDCl_3_) 77.0 ppm] for measuring chemical shifts δ in parts per million (ppm), J was measured in Hertz. The structure of the product was determined by means of ^1^H and ^13^C NMR spectra (Figures S1–S6). For the column chromatography, silica gel (60–200 mesh, Macherey-Nagel, Düren, Germany) was used. Eluent—chloroform:ethanol. HR-MS spectra were recorded at DFS Thermo Scientific spectrometer (Thermo Fisher Scientific, Waltham, MA, USA) in a full scan mode (15–500 *m/z*, 70 eV electron impact ionization, direct sample administration). Spectral and analytical measurements were carried out at the Multi-Access Chemical Service Center of Siberian Branch of Russian Academy of Sciences (SB RAS). Berberine chloride hydrate was purchased from TCI Co. (Tokyo, Japan) and used after drying in oven at 95 °C for 5 h. Pentylamine, hexylamine, and heptylamine were purchased from Acros Organics (Geel, Belgium) and used without additional purification.

General Procedure for the Synthesis of amines **3a**–**c**

Berberine chloride **1** (1 mmol) was treated with 5 mmol of alkylamine (pentylamine, hexylamine, and heptylamine, respectively). The reaction mixture was heated to 120–125 °C for 4–5 h. After completion, the reaction mixture was cooled to room temperature (20–23 °C) and diluted with acetone. The resulting precipitate was filtered, washed with a small amount of acetone, purified by column chromatography to give the compound **2a**–**c**. Amine **2a**–**c** was dissolved in methanol and treated with sodium borohydride in 0 °C. The reaction mixture was stirred for 30 min at 0 °C and for 3 h at room temperature (20–23 °C). Then, the solvent was removed under reduced pressure, the residue was dissolved in chloroform and washed with distilled water. The organic layer was separated from the water layer, dried with sodium sulfate, and evaporated at the rotary evaporator. After purification by column chromatography, the target compound **3a**–**c** was obtained.

9-(pentylamino)-2,3-methylenedioxy-10-methoxy-7,8,13,13a-tetrahydroprotoberberine chloride (**3a**)

Yield: 40%

^1^H NMR (400 MHz, CDCl_3_, δ): 6.72 (1H, s, H-11), 6.71 (2H, s, H-1, H-4), 6.57 (1H, s, H-12), 5.89 (2H, s, OCH_2_O), 4.12 (1H, d, J = 14.9 Hz, H-13a), 3.79 (3H, s, OCH_3_), 3.47–3.68 (3H, m, NH, H-8), 2.99–3.27 (4H, m, H-13, NH*CH_2_*), 2.79–2.91 (2H, m, H-5, H-6), 2.59–2.66 (2H, m, H-5, H-6), 1.48–1.56 (2H, m, *CH_2_*CH_2_CH_2_CH_3_)), 1.28–1.38 (4H, m, *CH_2_CH_2_*CH_3_), 0.89 (3H, tr, J = 7.1 Hz, CH_2_CH_2_*CH_3_*). ^13^C NMR (100 MHz, CDCl_3_, δ): 149.11 (C-3), 145.93, 145.70 (C-2, C-10), 135.11 (C-9), 130.90 (C-12a), 127.46, 127.19, 126.70 (C-4a, C-8a, C-13b), 121.28 (C-12), 108.97, 108.19 (C-4, C-11), 105.33 (C-1), 100.57 (OCH_2_O), 59.55 (C-13a), 55.78 (C-8), 55.71 (OCH_3_), 51.26 (C-6), 48.60 (NHCH_2_), 36.51 (C-13), 30.39 (NHCH_2_*CH_2_*CH_2_CH_2_CH_3_), 29.31, 29.05 (C-5, NHCH_2_CH_2_*CH_2_*CH_2_CH_3_), 22.39 (NHCH_2_CH_2_CH_2_*CH_2_*CH_3_), 13.95 (NHCH_2_CH_2_CH_2_CH_2_*CH_3_*). HR-MS, *m*/*z*: found 394.2253. Calculated for (C_24_H_30_O_3_N_2_)^+^: 394.2251.

9-(hexylamino)-2,3-methylenedioxy-10-methoxy-7,8,13,13a-tetrahydroprotoberberine chloride (**3b**)

Yield: 45%

^1^H NMR (400 MHz, CDCl_3_, δ): 6.71 (1H, s, H-11), 6.70 (2H, s, H-1, H-4), 6.57 (1H, s, H-12), 5.89 (2H, s, OCH_2_O), 4.11 (1H, d, J = 14.9 Hz, H-13a), 3.79 (3H, s, OCH_3_), 3.44–3.57 (2H, m, H-8), 2.97–3.27 (4H, m, H-13, NH*CH_2_*), 2.77–2.92 (2H, m, H-5, H-6), 2.55–2.68 (2H, m, H-5, H-6), 1.46–1.57 (2H, m, *CH_2_*CH_2_CH_2_CH_2_CH_3_), 1.23–1.39 (6H, m, *CH_2_CH_2_CH_2_*CH_3_), 0.87 (3H, tr, J = 6.8 Hz, CH_2_CH_2_*CH_3_*). ^13^C NMR (100 MHz, CDCl_3_, δ): 149.14 (C-3), 145.97, 145.72 (C-2, C-10), 135.15 (C-9), 131.05 (C-12a), 127.59, 127.32, 126.86 (C-4a, C-8a, C-13b), 121.33 (C-12), 109.01, 108.25 (C-4, C-11), 105.39 (C-1), 100.62 (OCH_2_O), 59.65 (C-13a), 55.90 (C-8), 55.78 (OCH_3_), 51.36 (C-6), 48.67 (NH*CH_2_*), 36.62 (C-13), 31.58 (NHCH_2_*CH_2_*CH_2_CH_2_CH_2_CH_3_), 30.71 (NHCH_2_CH_2_*CH_2_*CH_2_CH_2_CH_3_), 29.42, 26.57 (C-5, NHCH_2_CH_2_CH_2_*CH_2_*CH_2_CH_3_), 22.54 (NHCH_2_CH_2_CH_2_CH_2_*CH_2_*CH_3_), 13.95 (NHCH_2_CH_2_CH_2_CH_2_CH_2_*CH_3_*). HR-MS, *m*/*z*: found 408.2404. Calculated for (C_25_H_32_O_3_N_2_)^+^: 408.2407.

9-(heptylamino)-2,3-methylenedioxy-10-methoxy-7,8,13,13a-tetrahydroprotoberberine chloride (**3c**)

Yield: 25%

^1^H NMR (400 MHz, CDCl_3_, δ): 6.71 (1H, s, H-11), 6.70 (2H, s, H-1, H-4), 6.57 (1H, s, H-12), 5.89 (2H, s, OCH_2_O), 4.11 (1H, d, J = 15.1 Hz, H-13a), 3.79 (3H, s, OCH_3_), 3.44–3.70 (3H, m, NH, H-8), 2.97–3.27 (4H, m, H-13, NH*CH_2_*), 2.77–2.92 (2H, m, H-5, H-6), 2.55–2.68 (2H, m, H-5, H-6), 1.47–1.54 (2H, m, *CH_2_*CH_2_CH_2_CH_2_CH_2_CH_3_), 1.23–1.37 (8H, m, *CH_2_CH_2_CH_2_CH_2_*CH_3_), 0.86 (3H, tr, J = 6.6 Hz, CH_2_CH_2_*CH_3_*). ^13^C NMR (100 MHz, CDCl_3_, δ): 149.10 (C-3), 145.93, 145.68 (C-2, C-10), 135.12 (C-9), 131.04 (C-12a), 127.56, 127.30, 126.85 (C-4a, C-8a, C-13b), 121.29 (C-12), 108.96, 108.21 (C-4, C-11), 105.35 (C-1), 100.58 (OCH_2_O), 59.62 (C-13a), 55.88 (C-8), 55.74 (OCH_3_), 51.34 (C-6), 48.62 (NH*CH_2_*), 36.61 (C-13), 31.70, 30.73 (NHCH_2_*CH_2_*CH_2_CH_2_CH_2_CH_2_CH_3_, NHCH_2_CH_2_*CH_2_*CH_2_CH_2_CH_2_CH_3_), 29.41, 29.01, 26.83 (C-5, NHCH_2_CH_2_CH_2_*CH_2_*CH_2_CH_2_CH_3_, NHCH_2_CH_2_CH_2_CH_2_*CH_2_*CH_2_CH_3_), 22.48 (NHCH_2_CH_2_CH_2_CH_2_CH_2_*CH_2_*CH_3_), 13.97 (NHCH_2_CH_2_CH_2_CH_2_CH_2_CH_2_*CH_3_*). HR-MS, *m*/*z*: found 422.2568. Calculated for (C_26_H_34_O_3_N_2_)^+^: 422.2564.

### 4.2. Biological Experiments

#### 4.2.1. Animals

The study involved male C57BL/6 mice weighing 23–25 g and male AY mice weighing 28–32 g. Animals were obtained from the SPF vivarium of the Institute of Cytology and Genetics SB RAS. The animals were housed in polycarbonate cages with ad libitum access to water and feed. In the rooms of vivarium humidity, temperature and 12/12 h light-and-dark cycle were controlled. All manipulations with animals were carried out in strict accordance with the laws of the Russian Federation, a decree of the Ministry of Health of the Russian Federation no. 199n of 4 January 2016, and Directive 2010/63/EU of the European Parliament and of the Council of the European Union of 22 September 2010 on the protection of animals used for scientific purposes. The protocol of the animal experiment was approved by the Ethics Committee of N.N. Vorozhtsov Institute of Organic Chemistry SB RAS (protocol no. P-01-04.2022-14). During the experiment, the mice’s body mass and food consumption were evaluated once a week.

#### 4.2.2. The OGTT

The test was performed on C57BL/6 mice for screening purposes and on AY during long-term experiment (*n* = 6 in each group) after a 12 h fasting. Oral glucose loading (2.5 g/kg) was done in all groups of mice. Prior to dissolution in water, compounds **3a**–**c** were mixed with two drops of Tween 80 and administered at a dose of 5 (compound **3c**), 15 (all), 30 (all), and 45 (compound **3c**) mg/kg. Vildagliptin (VLD) tablets (Galvus, Novartis Farmaceutica SA, Barcelona, Spain) were dissolved in water and used as the positive control at a dose of 10 mg/kg in the OGTT in C57BL/6 mice. In the case of the AY mice experiment, a water solution of metformin (MF, CAS 1115-70-4 Acros Organics, Geel, Belgium) at a dose of 250 mg/kg was used as the positive control according to the experimental design (Section 4.2.4). At the 14 days’ time point, all compounds were introduced 30 min prior to the glucose load by oral gavage; at the 28 days’ time point, the last compounds’ introduction was a day prior to test. In all tests, blood samples were obtained from tail incision before dosing (time 0) and at 30, 60, 90, and 120 min after the glucose load. Blood glucose concentration was evaluated with a ONE TOUCH Select blood glucose meter (LIFESCAN Inc., Milpitas, CA, USA). The area under the glycemic curve (AUC) was calculated using Tai’s model [33].

#### 4.2.3. The ITT

The test was performed on all animals according to the experimental design (Section 4.2.4) after a 4 h fasting. Compounds were introduced by oral gavage 4 h prior to insulin injection. Insulin (Soluble human insulin, Medsynthesis plant, Novouralsk, Russia) was injected i.p. at a dose of 5 ED/kg. Blood samples were obtained from tail incision before dosing (time 0) and at 15, 30, 45, 60, and 90 min after the insulin injection. Blood glucose concentration was evaluated with a ONE TOUCH Select blood glucose meter (LIFESCAN Inc., Milpitas, CA, USA).

#### 4.2.4. The Design of the Experiment on AY Mice

In order to facilitate the body weight gain, mice were fed standard chow plus lard and cookies ad libitum for 30 days. Animals with body weight more than 35 g were selected for the experiment and divided in the following groups: (1) AY mice (*n* = 6) + vehicle (water + 2 drops of Tween 80), (2) AY mice (*n* = 6) + compound **3c** 15 mg/kg, (3) AY mice (*n* = 6) + MF 250 mg/kg, and (4) C57BL/6 mice (*n* = 6) + vehicle (water + 2 drops of Tween 80). The animals were on the same diet till the end of the experiment. All compounds were given once a day by oral gavage. OGTT was performed on the 28th day of the experiment. ITT was performed on the 29th day of the experiment. On day 31, animals were decapitated, and blood was drawn for the biochemical assay, insulin, and adiponectin measurement. Liver, interscapular white and brown fat, and pancreas were excised for histological evaluation and ELISA examination (liver only). In case of C57Bl/6 mice, gonadal white fat was taken instead of interscapular.

#### 4.2.5. ELISA Examination

To analyze insulin and adiponectin serum concentrations, glucokinase level in liver homogenates ELISA examinations and sample preparation were conducted according to the manufacturer’s instruction manual (Cloud-Clone Corp., Katy, TX, USA). Homogenization was done using Q125 Sonicator (Qsonica, Newtown, CT, USA). Multiscan Ascent (Thermo Labsystems, Helsinki, Finland) photometer was used for analysis.

#### 4.2.6. Biochemical Assays

After 31 days of treatment, mice were decapitated, blood was collected, and serum was separated by centrifugation at 1640× *g* for 15 min. Serum total cholesterol, triglycerides, total protein, alkaline phosphatase, alanine aminotransferase, and lactate levels were quantified in all groups using standard diagnostic kits (Vector-Best, Novosibirsk, Russia) and a Stat Fax 3300 spectrophotometer (Awareness Technology Inc., Palm City, FL, USA).

#### 4.2.7. Histological Examination

Liver, interscapular brown fat, and pancreas were fixed in 10% neutral buffered formalin for 7 days, then standard dehydration in ascending ethanol concentrations and xylene was carried out. All samples were embedded in paraffin on an AP 280 workstation using Histoplast (Thermo Fisher Scientific, Waltham, MA, USA) with a melting point of 58 °C. Tissues were sliced with a thickness of 4.5 μm on a rotational microtome NM 335E with disposable interchangeable blades. The slices were stained with periodic acid–Schiff, hematoxylin and eosin, and orange G and examined under a light microscope at a magnification of ×100–200.

#### 4.2.8. Statistical Analysis

Statistical analysis was performed by the Mann–Whitney U test. Data are shown as mean ± SEM. Data with *p* < 0.05 were considered statistically significant.

## 5. Conclusions

Three 9-N-alkyltetrahydroberberines were synthesized among which one, compound **3c**, containing a 7-membered aliphatic substituent, was selected based on the OGTT results for study in obese mice with impaired carbohydrate metabolism (C57Bl/6^Ay^) under four-week oral administration. These studies found a significant hypoglycemic effect associated with increased insulin sensitivity in mice. Long-term administration of compound **3c** did not reveal its toxic effect on animals, as evidenced by body weight, feed intake, biochemical parameters, and histological data.

## Figures and Tables

**Table 1:** Evaluation of compounds **3a**–**c** in OGTT. Data represented as a difference of area under the glycemic curve (AUC) in % from control mice (glucose load only) in the relevant experiment. Each group *n* = 6. n.d.—no data.

Dose	5 mg/kg		15 mg/kg		30 mg/kg		45 mg/kg	
Group	Effect	Mortality	Effect	Mortality	Effect	Mortality	Effect	Mortality
**3a**	0	0	0	0	0	0	n.d.	n.d.
**3b**	0	0	9.4	0	43.1	33.3	n.d.	n.d.
**3c**	0	0	24.5	0	13.9	0	26	16.6

**Table 2:** Food consumption by mice during the experiment. Data represented as food intake (total amount of standard chow, cookies, and lard) in g per a mouse in the group.

Experiment Week	3c	MF	AY
	g/Mouse		
1	25.4	29.9	23.7
2	27.6	29.8	26.2
3	23.8	25.9	25.3
4	26.3	26.2	23.8

**Table 3:** Mass of livers, gonadal fat pads, interscapular fat pads, and brown fat in AY mice treated for four weeks by compound **3c** at a dose of 15 mg/kg and MF at a dose of 250 mg/kg. * *p* < 0.05 as compared to AY mice.

	Body Mass, g	Liver Mass, g	Gonadal Fat Pad, g	Interscapular Fat Pad, g	Interscapular Brown Fat, g
C57Bl/6	21.90 ± 0.30 *	0.85 ± 0.013 *	0.27 ± 0.02 *	-	0.06 ± 0.003 *
AY	36.97 ± 2.41	1.09 ± 0.05	1.99 ± 0.26	1.10 ± 0.16	0.20 ± 0.009
**3c**	36.37 ± 2.12	1.06 ± 0.04	2.01 ± 0.21	1.01 ± 0.14	0.15 ± 0.02 *
MF	32.45 ± 2.01	1.07 ± 0.06	1.93 ± 0.26	0.73 ± 0.12	0.13 ± 0.02 *

**Table 4:** Biochemical parameters of mouse blood. TC—total cholesterol, TG—triglycerides, TP—total protein, ALT—alanine aminotransferase, AST—aspartate transaminase. * *p* < 0.05 as compared to AY mice.

Group	TC, mmol/L	TG, mmol/L	TP, g/L	Lactate, mmol/L	ALT, U/L	AST, U/L
C57Bl/6	4.11 ± 0.03	1.37 ± 0,02 *	6.79 ± 0.14	8.94 ± 0.29	13.39 ± 3.36	45.39 ± 2.96
AY	4.03 ± 0.05	1.54 ± 0.03	6.37 ± 0.46	8.63 ± 0.11	20.25 ± 3.10	46.85 ± 2.53
**3c**	3.96 ± 0.45	1.57 ± 0.02	6.66 ± 0.09	7.94 ± 0.28 *	19.21 ± 2.69	45.15 ± 4.43
MF	4.48 ± 0.48	1.53 ± 0.09	6.77 ± 0.13	7.93 ± 0.24 *	15.71 ± 1.50	48.89 ± 3.37

## Data Availability

Not applicable.

## Supplementary Materials

The supporting information can be downloaded at: https://www.mdpi.com/article/10.3390/ijms232214186/s1.

## References

[1] DeFronzo R.A., Ferrannini E., Groop L., Henry R.R., Herman W.H., Holst J.J., Hu F.B., Kahn C.R., Raz I., Shulman G.I. (2015). Type 2 diabetes mellitus. Nat. Rev. Dis. Prim..

[2] Guo W., Liu J., Cheng L., Liu Z., Zheng X., Liang H., Xu F. (2021). Metformin Alleviates Steatohepatitis in Diet Induced Obese Mice in a SIRT1-Dependent Way. Front. Pharmacol..

[3] McCreight L.J., Bailey C.J., Pearson E.R. (2016). Metformin and the gastrointestinal tract. Diabetologia.

[4] American Diabetes Association (2021). 9. Pharmacologic Approaches to Glycemic Treatment: Standards of Medical Care in Diabetes. Diabetes Care.

[5] Gray A., Threlkeld R.J., Feingold K.R., Anawalt B., Boyce A., Chrousos G., de Herder W.W., Dhatariya K., Dungan K., Hershman J.M., Hofland J., Kalra S. (2019). Nutritional Recommendations for Individuals with Diabetes. Endotext [Internet].

[6] Kong Y., Li L., Zhao L., Yu P., Li D. (2022). A patent review of berberine and its derivatives with various pharmacological activities. Expert Opin. Ther. Pat..

[7] Albarrati A., Albratty M., Behl T., Bungau S., Meraya A.M., Najmi A., Sharma N., Singh S., Zahoor I. (2022). Expatiating the Pharmacological and Nanotechnological Aspects of the Alkaloidal Drug Berberine: Current and Future Trends. Molecules.

[8] Xing Y., Liu X., Lin Y., Zhang Y. (2017). Progress in pharmacological effects and clinical applications of berberine. Chin. J. Pharmacol. Toxicol..

[9] Pang B., Zhao L.-H., Zhou Q., Zhao T.-Y., Wang H., Gu C.-J., Tong X.-L. (2015). Application of Berberine on Treating Type 2 Diabetes Mellitus. Int. J. Endocrinol..

[10] Pirillo A., Catapano A.L. (2015). Berberine, a plant alkaloid with lipid- and glucose-lowering properties: From in vitro evidence to clinical studies. Atherosclerosis.

[11] Zhao J., Wang Z., Karrar E., Xu D., Sun X. (2022). Inhibition Mechanism of Berberine on α-Amylase and α-Glucosidase in Vitro. Starch.

[12] Zhao Y., Li Z., Lu E., Sheng Q., Zhao Y. (2021). Berberine exerts neuroprotective activities against cerebral ischemia/reperfusion injury through up-regulating PPAR-γ to suppress NF-κB-mediated pyroptosis. Brain Res. Bull..

[13] Zhou J., Zhou S. (2010). Berberine regulates peroxisome proliferator-activated receptors and positive transcription elongation factor b expression in diabetic adipocytes. Eur. J. Pharm..

[14] Jiang S.-J., Dong H., Li J.-B., Xu L.-J., Zou X., Wang K.-F., Lu F.-E., Yi P. (2015). Berberine inhibits hepatic gluconeogenesis *via* the LKB1-AMPK-TORC2 signaling pathway in streptozotocin-induced diabetic rats. World J. Gastroenterol..

[15] Sun R., Yang N., Kong B., Cao B., Feng D., Yu X., Ge C., Huang J., Shen J., Wang P. (2017). Orally Administered Berberine Modulates Hepatic Lipid Metabolism by Altering Microbial Bile Acid Metabolism and the Intestinal FXR Signaling Pathway. Mol. Pharmacol..

[16] Sun R., Kong B., Yang N., Cao B., Feng D., Yu X., Ge C., Feng S., Fei F., Huang J. (2021). The Hypoglycemic Effect of Berberine and Berberrubine Involves Modulation of Intestinal FXR Signaling Pathway and Inhibition of Hepatic Gluconeogenesis. Drug Metab. Dispos..

[17] Xu G., Wan H., Yi L., Chen W., Luo Y., Huang Y., Liu X. (2021). Berberine administrated with different routes attenuates inhaled LPS-induced acute respiratory distress syndrome through TLR4/NF-κB and JAK2/STAT3 inhibition. Eur. J. Pharmacol..

[18] Li W., Hua B., Saud S.M., Lin H., Hou W., Matter M.S., Jia L., Colburn N.H., Young M.R. (2015). Berberine regulates AMP-activated protein kinase signaling pathways and inhibits colon tumorigenesis in mice. Mol. Carcinog..

[19] Turner N., Li J.-Y., Gosby A., To S.W.C., Cheng Z., Miyoshi H., Taketo M.M., Cooney G.J., Kraegen E.W., James D.E. (2008). Berberine and its more biologically available derivative, dihydroberberine, inhibit mitochondrial respiratory complex I: A mechanism for the action of berberine to activate AMP-activated protein kinase and improve insulin action. Diabetes.

[20] Chen C., Zhang Y., Huang C. (2010). Berberine inhibits PTP1B activity and mimics insulin action. Biochem. Biophys. Res. Commun..

[21] Chen W., Miao Y.-Q., Fan D.-J., Yang S.-S., Lin X., Meng L.-K., Tang X. (2011). Bioavailability Study of Berberine and the Enhancing Effects of TPGS on Intestinal Absorption in Rats. AAPS Pharmscitech..

[22] Xia X., Yan J., Shen Y., Tang K., Yin J., Zhang Y., Yang D., Liang H., Ye J., Weng J. (2011). Berberine Improves Glucose Metabolism in Diabetic Rats by Inhibition of Hepatic Gluconeogenesis. PLoS ONE.

[23] Gaba S., Monga V., Saini A., Singh G. (2021). An insight into the medicinal attributes of berberine derivatives: A review. Bioorg. Med. Chem..

[24] Nowak R., Och A., Och M., Podgórska D., Podgórski R. (2022). Berberine, a Herbal Metabolite in the Metabolic Syndrome: The Risk Factors, Course, and Consequences of the Disease. Molecules.

[25] Singh N., Sharma B. (2018). Toxicological Effects of Berberine and Sanguinarine. Front. Mol. Biosci..

[26] Zhang S., Wang X., Yin W., Liu Z., Zhou M., Xiao D., Liu Y., Peng D. (2016). Synthesis and hypoglycemic activity of 9-O-(lipophilic group substituted) berberine derivatives. Bioorg. Med. Chem. Lett..

[27] Khvostov M.V., Gladkova E.D., Borisov S.A., Zhukova N.A., Marenina M.K., Meshkova Y.V., Luzina O.A., Tolstikova T.G., Salakhutdinov N.F. (2021). Discovery of the First in Class 9-N-Berberine Derivative as Hypoglycemic Agent with Extra-Strong Action. Pharmaceutics.

[28] Qi Y., Takahashi N., Hileman S.M., Patel H.R., Berg A.H., Pajvani U.B., Scherer P.E., Ahima R.S. (2004). Adiponectin Acts in the Brain to Decrease Body Weight. Nat. Med..

[29] Lovejoy J., Newby F., Gebhart S., DiGirolamo M. (1992). Insulin resistance in obesity is associated with elevated basal lactate levels and diminished lactate appearance following intravenous glucose and insulin. Metabolism.

[30] Massa M.L., Gagliardino J.J., Francini F. (2011). Liver glucokinase: An overview on the regulatory mechanisms of its activity. IUBMB Life.

[31] Miranda C.S., Silva-Veiga F., Martins F.F., Rachid T.L., Mandarim-De-Lacerda C.A., Souza-Mello V. (2020). PPAR-α activation counters brown adipose tissue whitening: A comparative study between high-fat- and high-fructose-fed mice. Nutrition.

[32] Sharma B., Xie L., Yang F., Wang W., Zhou Q., Xiang M., Zhou S., Lv W., Jia Y., Pokhrel L. (2020). Recent Advance on PTP1B Inhibitors and Their Biomedical Applications. Eur. J. Med. Chem..

[33] Tai M.M. (1994). A Mathematical Model for the Determination of Total Area Under Glucose Tolerance and Other Metabolic Curves. Diabetes Care.

