# Supplementary material for: Study of Hypoglycemic Activity of Novel 9-N-alkyltetrahydroberberine Derivatives
Source: Int J Mol Sci. 2022 Nov 16;23(22):14186. doi: 10.3390/ijms232214186 (PMC9698964; doi:10.3390/ijms232214186)

SUPPLEMENTARY MATERIALS

Study of hypoglycemic activity of novel 9-N-alkyltetrahydroberberine derivatives.

Mikhail V. Khvostov [1], Elizaveta D. Gladkova [1], Sergey A. Borisov [1], Marina S. Fedotova [1,2], Nataliya A. Zhukova [1], Mariya K. Marenina [1], Yuliya V. Meshkova [1], Olga A. Luzina [1,*], Tatiana G. Tolstikova [1] and Nariman F. Salakhutdinov [1]

1 N. N. Vorozhtsov Novosibirsk Institute of Organic Chemistry, Siberian Branch of the Russian Academy of Sciences, 9, Akademika Lavrentieva Ave., 630090 Novosibirsk, Russia;

2 Department of Medical Chemistry, Novosibirsk State University, Pirogova Str. 1, 630090 Novosibirsk, Russia

* Correspondence: luzina@nioch.nsc.ru

NMR ^{1}H and ^{13}C spectra of the compounds **3a – 3c**

Figure S1. Compound **3a** (¹H NMR, solvent – $CDCl_3$)

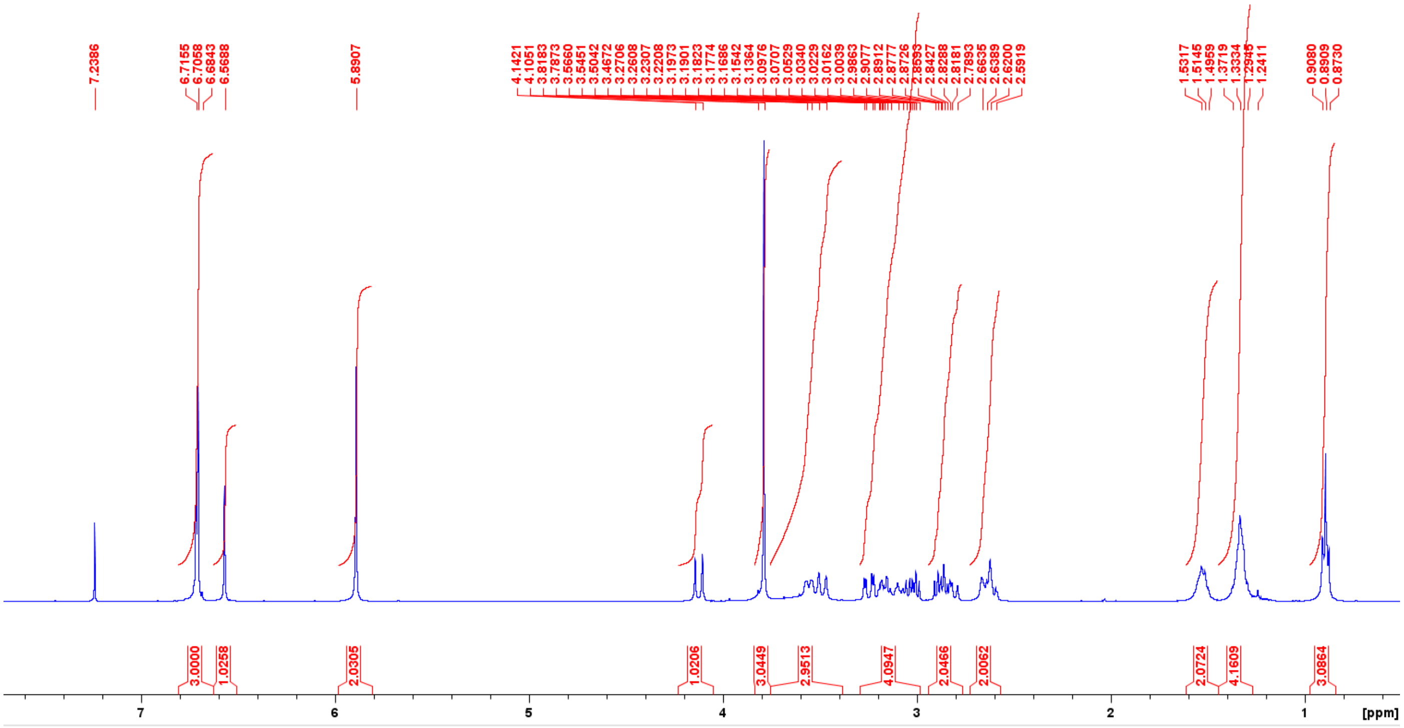

Figure S2. Compound **3a** (¹³C NMR, solvent – $CDCl_3$)

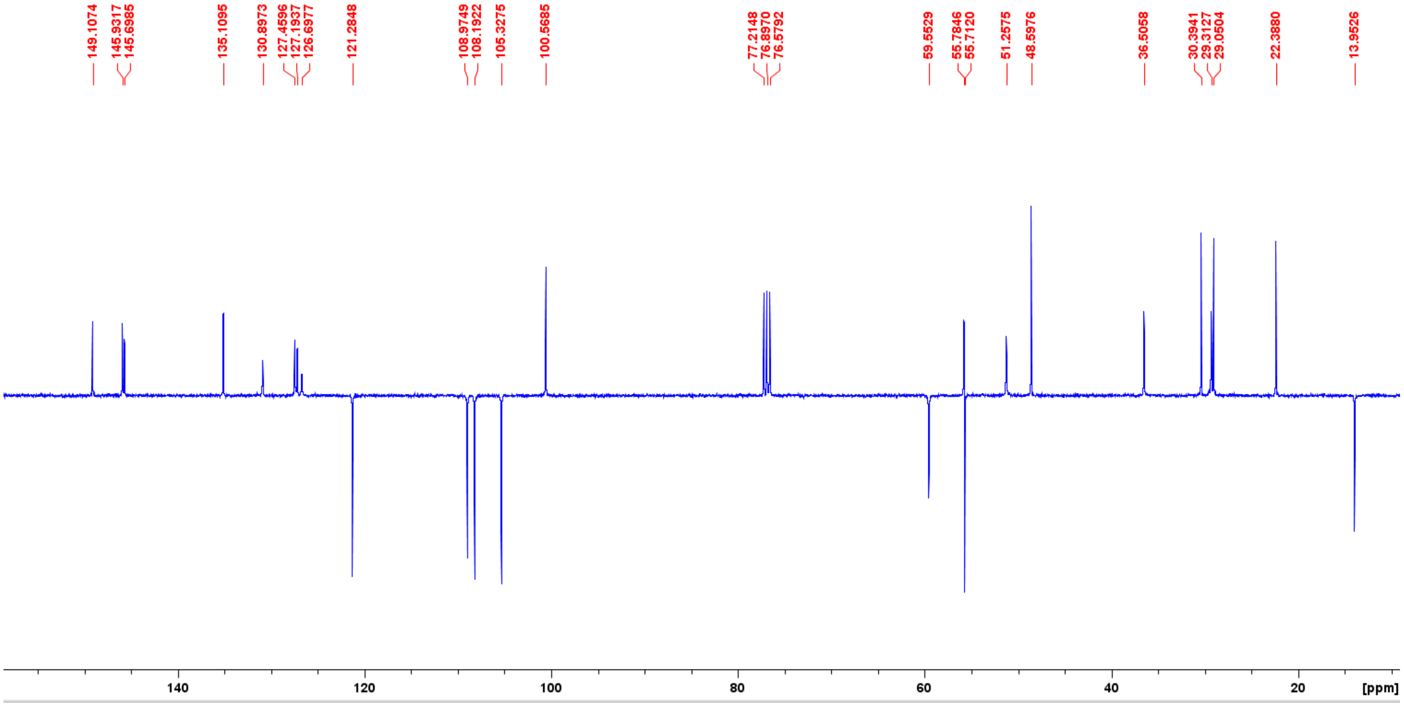

Figure S3. Compound **3b (^{1}H NMR, solvent – $CDCl_3$)**

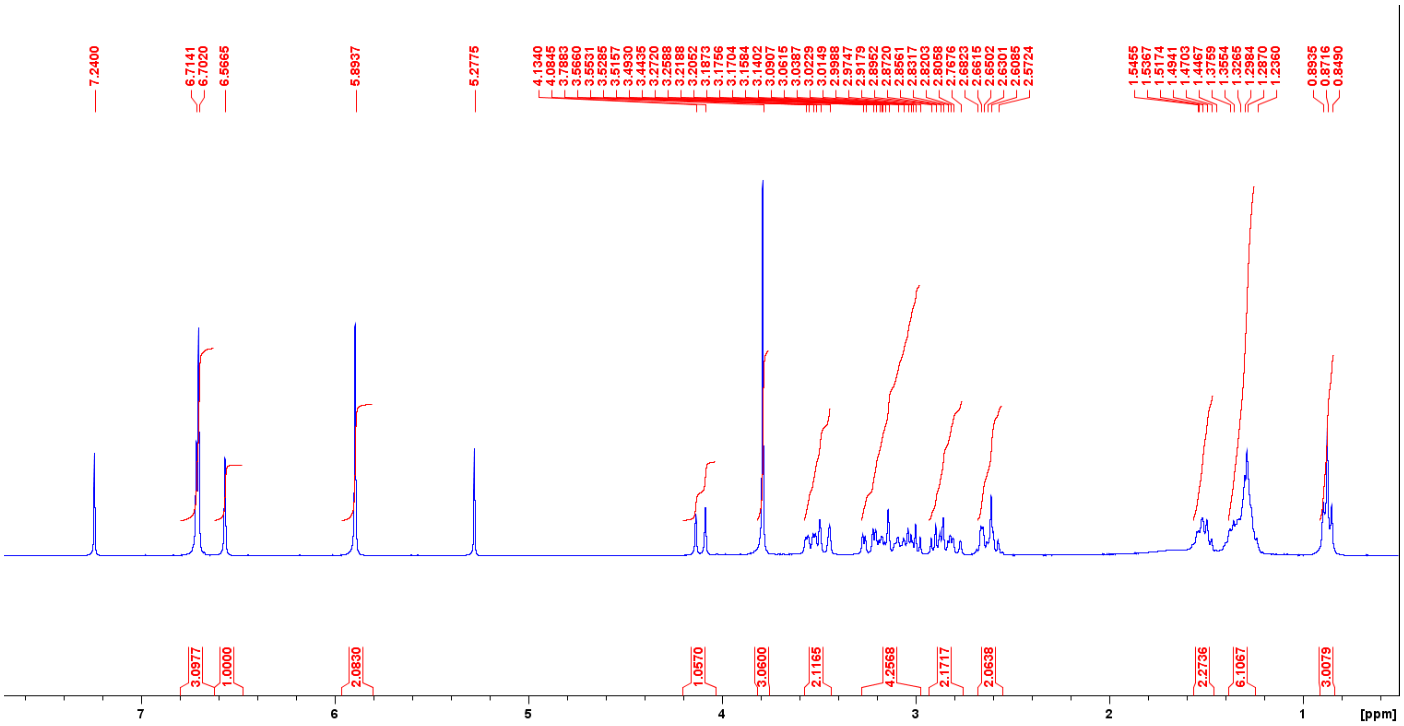

Figure S4. Compound **3b (^{13}C NMR, solvent – $CDCl_3$)**

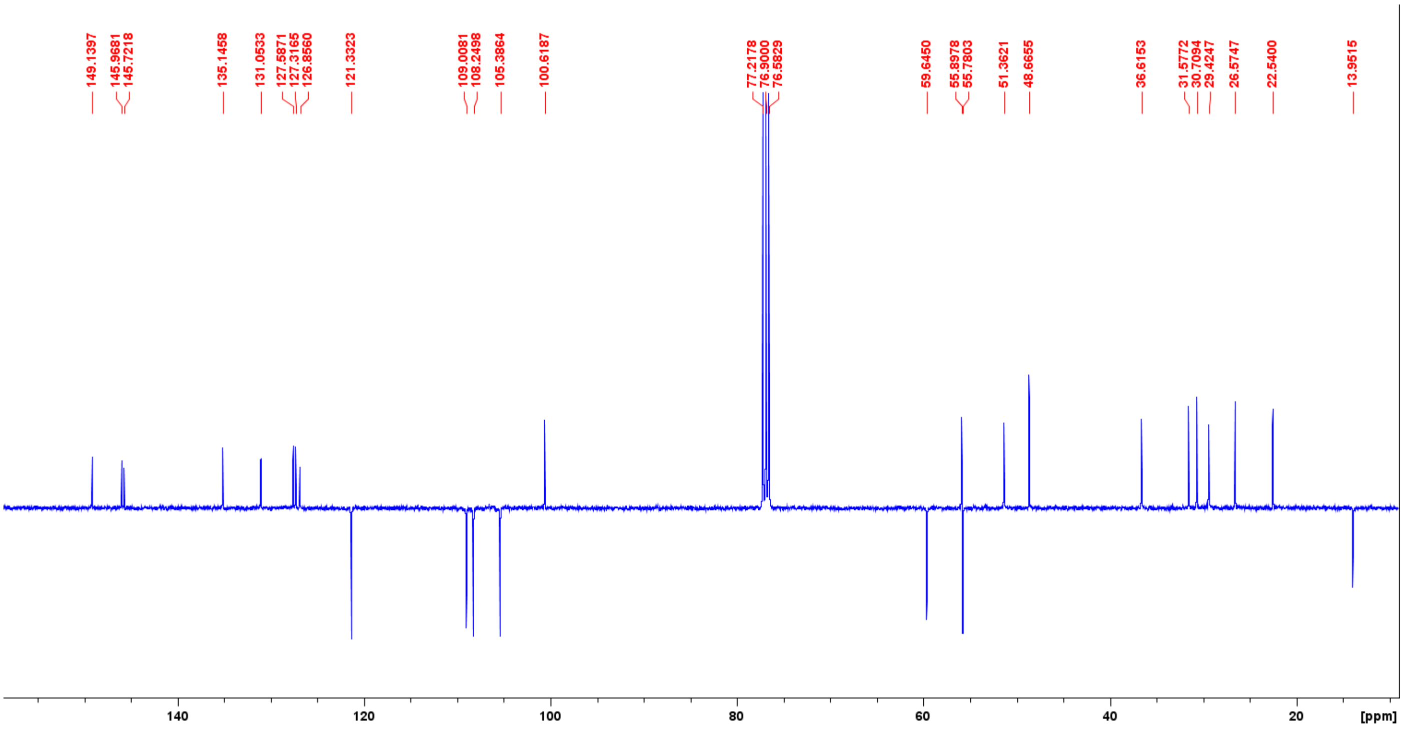

Figure S5. Compound **3c** (^{1}H **NMR, solvent – $CDCl_3$**)

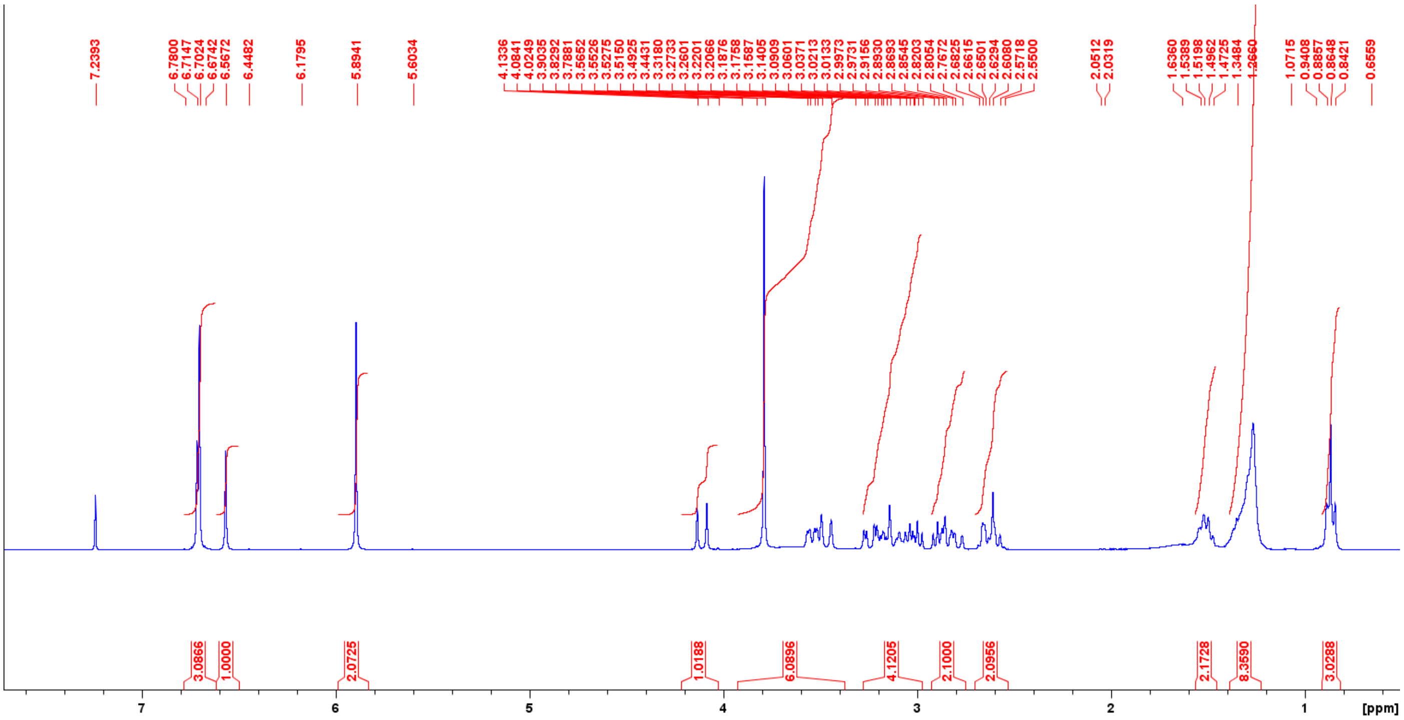

Figure S6. Compound **3c** (^{13}C **NMR, solvent – $CDCl_3$**)

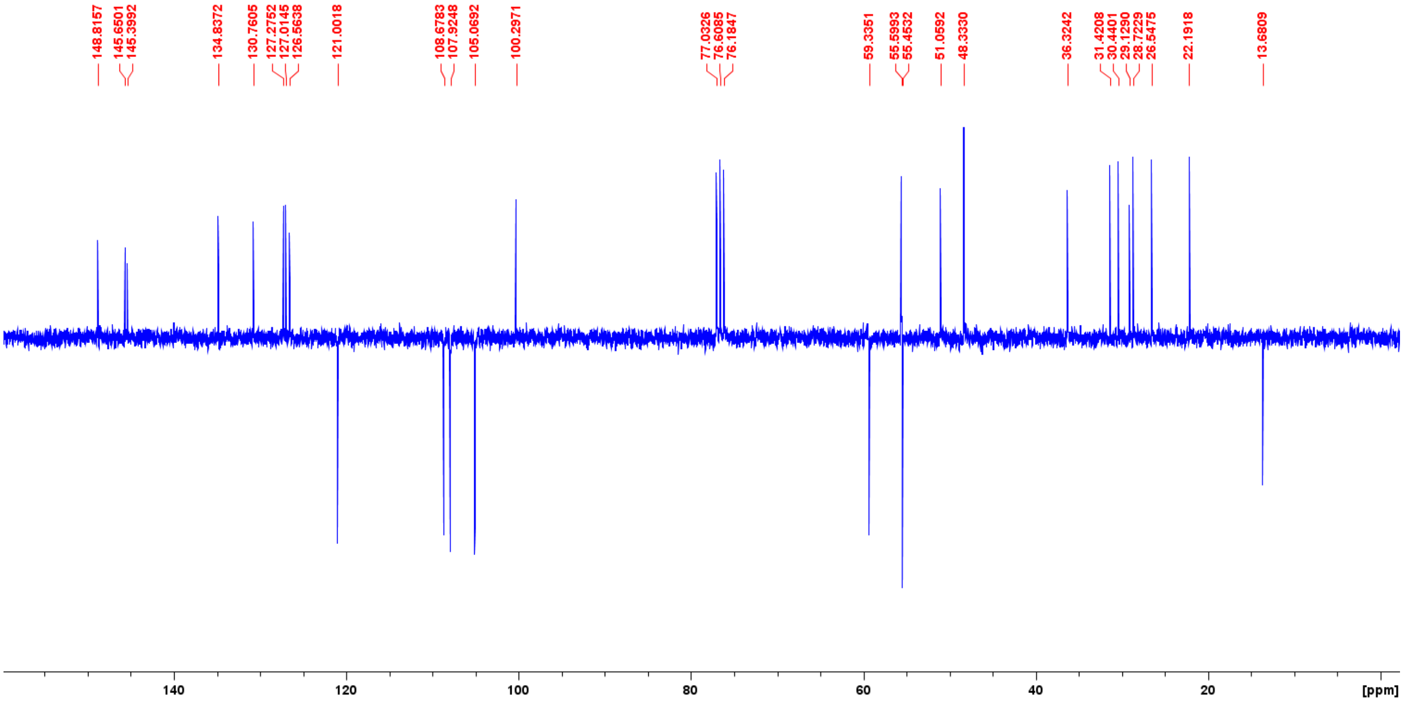

Supplement: Supplementary file 1 [file ijms-23-14186-s001.zip › ijms-2020824-supplementary.pdf]
